# Unusual interaction of RNA polymerase with the bacteriophage Mu middle promoter P_m_ in the absence of its activator protein Mor

**DOI:** 10.1002/mbo3.181

**Published:** 2014-06-10

**Authors:** Yongkai Mo, Martha M Howe

**Affiliations:** 1 Department of Microbiology, Immunology and Biochemistry, University of Tennessee Health Science Center858 Madison Ave., Memphis, Tennessee, 38163

**Keywords:** Bacteriophage Mu, Mor activator protein, Mu middle promoter P_m_, prokaryotic transcription, RNAP-promoter interactions

## Abstract

The bacteriophage Mu Mor activator protein is absolutely required for transcription from the Mu middle promoter P_m_. However, when RNA polymerase (RNAP) was incubated with P_m_ DNA in the absence of Mor, a band at promoter position −51 was hypersensitive to DNase I cleavage, demonstrating an interaction of RNAP with the promoter DNA. The hypersensitivity was similar at four different lengths of P_m_ DNA assayed from −62 to +10, −62 to +46, −96 to +10, and −96 to +46. The hypersensitivity occurred equally well at 5°C, 15°C, and 30°C, indicating that it did not require open complex formation, which only occurred at 30°C. The −51 hypersensitivity at 5°C and 15°C was eliminated by the addition of heparin, consistent with the possibility that it arose by formation of unstable closed complexes of RNAP bound to P_m_ DNA. Generation of the hypersensitive band required the complete RNAP with its *α*CTDs, but neither the *α*CTD nor intact *α* were sufficient for the interaction and resulting hypersensitivity. There was no correlation between the level of hypersensitivity observed in vitro and the level of P_m_ activity in vivo*,* as assayed by the Mor-dependent production of *β*-galactosidase from a P_m_-*lacZ* fusion. In an “order of addition” experiment, preincubation of P_m_ DNA with Mor followed by addition of RNAP led to the fastest open complex formation, whereas preincubation of P_m_ DNA with RNAP gave the slowest. These results support the conclusion that Mor recruits RNAP to P_m_ rather than reposition a prebound RNAP, as occurs for C-dependent repositioning of RNAP at the Mu late promoter P_mom_.

## Introduction

The DNA-dependent RNA polymerase (RNAP) of *Escherichia coli* K-12 (*E. coli*) is the key enzyme involved in gene expression. It performs multiple functions in transcrip-tion including promoter recognition, abortive initiation, promoter clearance, elongation, pausing, and termination. Each of these steps can involve conformational changes and serve as a substrate for subsequent steps and thus are targets for regulation (for reviews see articles by Gourse et al. 2000; Ishihama 2000; Erie 2002; Hsu 2002; Young et al. 2002; Murakami and Darst 2003; Browning and Busby 2004; Landick 2006). The RNAP core enzyme contains five subunits—two *α* subunits, *β*, *β*′, and *ω*—and is capable of catalyzing nonspecific RNA synthesis. Promoter-specific initiation of transcription is conferred by addition of the *σ* subunit to form the RNAP holoenzyme (*α*_2_*ββ*′*ωσ*). There are multiple *σ* factors which direct transcription to different sets of genes by recognizing and binding to different specific promoter sequences, allowing the cell to respond to varying environmental conditions and physiological needs (Ishihama 2000). The 613 amino acid *σ*^70^ subunit is by far the most abundant *σ* factor in *E. coli* and is used for transcription of the majority of *E. coli* genes (Ishihama 2000); because of its broad use, it is often referred to as the “housekeeping” *σ* factor. RNAP holoenzyme containing *σ*^70^ recognizes two hexameric sequences located in the −10 and −35 regions, which comprise the core promoter; these hexamers are bound by *σ*^70^ regions 2.4 and 4.2, respectively (Borukhov and Severinov 2002; Young et al. 2002). The *α* subunit consists of 329 amino acids that fold into a structure with two independently folded domains connected by a protease-sensitive flexible linker (Negishi et al. 1995). Each domain is responsible for distinct functions. The *α* N-terminal domain (*α*NTD) plays an essential role in RNAP assembly by providing the contact surfaces for *α* dimerization and binding of the *β* and *β*′ subunits (Kimura and Ishihama 1995). The *α* C-terminal domain (*α*CTD) plays a regulatory role by binding to AT-rich UP elements located upstream of the −35 region in some promoters and by providing contact surfaces for interaction with *trans*-acting protein factors called activators (Ishihama 1992; Gaal et al. 1996; Gourse et al. 2000). The large *β* and *β*′ subunits (1342 and 1407 amino acids, respectively) make up the majority of the catalytic site as well as the two sides of the crab claw structure that closes around the template DNA (Zhang et al. 1999; Murakami et al. 2002a,b). The 91 amino acid *ω* subunit is involved in RNAP assembly and stability (Minakhin et al. 2001).

Transcription initiation in prokaryotes can be divided into at least four distinct phases: RNAP binding, isomerization, abortive initiation, and promoter clearance (deHaseth and Helmann 1995; Saecker et al. 2011). First, RNAP holoenzyme binds to the promoter and forms one or more closed complexes (RP_c_) that are in rapid equilibrium with free promoter DNA and free RNAP (McClure 1985; Cowing et al. 1989; Mecsas et al. 1991). In the closed complex RNAP covers one face of the double-stranded DNA helix (Schickor et al. 1990), which is partially wrapped around RNAP (Coulombe and Burton 1999; Murakami et al. 2002a,b). The closed complex typically has a DNase I footprint that extends roughly from base −60 to base −5 (Kovacic 1987; Cowing et al. 1989; Schickor et al. 1990). Heparin, as a DNA mimic, competes with promoter DNA for RNAP binding and is used to eliminate closed complexes (McClure 1985). Isomerization includes at least two steps. In the first step, RP_c_ is converted to one or more intermediate complexes (RP_i_) which exhibit footprints that extend downstream to base +15 to +20 (Cowing et al. 1989; Mecsas et al. 1991; Craig et al. 1995), suggesting more extensive wrapping of DNA around RNAP (Coulombe and Burton 1999; Rivetti et al. 1999). During formation of RP_i_ there is a significant conformational change, resulting in substantial DNA untwisting but no DNA strand separation (Cowing et al. 1989; Mecsas et al. 1991). In the second step, the torsional stress is relieved by DNA melting that extends downstream from within the −10 hexamer to just beyond the transcription start site at +1, generating one or more open complexes (RP_o_) (Cowing et al. 1989; Schickor et al. 1990; Mecsas et al. 1991). Typically, open complexes are stable and resistant to heparin challenge (McClure 1985). Open complex formation generally requires temperatures of 25°C or higher and is inhibited at temperatures less than 20°C (Cowing et al. 1989; Schickor et al. 1990; Mecsas et al. 1991; Coulombe and Burton 1999). In the presence of NTPs and ATP, open complexes initiate transcription, resulting in the synthesis of short transcripts (<12 nt), most of which are released, a process termed abortive initiation (Munson and Reznikoff 1981). During this process, RNAP remains bound to the promoter (Krummel and Chamberlin 1989). Once RNAP synthesizes a sufficiently long transcript (∼8 to 12 nt), the polymerase escapes from the promoter, releasing the *σ* subunit and forming a stable elongation complex (Hsu 2002).

Many bacterial genes with poor −35 hexamers (match 3 or 4 bases of the −35 consensus sequence TTGACA) are up-regulated by activator protein binding to the promoter and interacting with RNAP (Ptashne and Gann 1997; Hochschild and Dove 1998; Salgado et al. 2013). Any subunit of RNAP can serve as a contact site for an activator and any of the four phases of transcription initiation can be affected (Browning and Busby 2004; Ishihama 2010). Nevertheless, many of the activators can be divided into two groups based on the binding site and the RNAP subunit contacted. For Class I activators, the binding site is in the −60 region and the *α*CTD is contacted. For those in Class II, the activator generally binds in the −40 region and contacts the *σ*CTD. However, there are a significant number of activators which do not fit into either of these two classes. Likewise, the activation mechanism varies from one activator and promoter to another. For example, the activator CAP stimulates *lac* operon transcription by binding to the *lac* promoter and using contacts with the *α*CTD to recruit RNAP to bind (Malan et al. 1984; Gourse et al. 2000). The *λ*cI activator stimulates transcription at P_RM_ by increasing the isomerization rate of prebound RNAP (Li et al. 1994). At the *gal*P1 promoter CAP stimulates transcription by increasing both recruitment and the rate of isomerization (Belyaeva et al. 1996; Niu et al. 1996). Finally, at the *malT* promoter, CAP accelerates the escape of RNAP from the initiation complex (Eichenberger et al. 1997). Other examples can be found in the review by Browning and Busby (2004).

**Figure 1:**
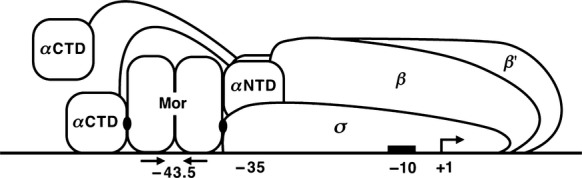
Model for P_m_ promoter initiation complex. The horizontal line represents P_m_ DNA with relevant positions marked below it. Mor is shown as a dimer bound to the dyad-symmetric element indicated by inverted arrows centered at −43.5. The −10 hexamer is shown as a black box, and the transcription start (+1) is designated by a bent arrow. The Mor-*α*CTD and Mor-*σ*CTD interactions are designated by black ovals. The RNAP subunits are labeled; the position of the second *α*CTD is not known. This figure is based on observations of the Mor-RNAP interaction paper from the Howe lab (Artsimovitch et al. 1996).

Bacteriophage Mu is a temperate phage that infects *E. coli* K-12 and multiple species of other enteric bacteria (for reviews, see Howe 1998; Howe and Pato 2013; Paolozzi and Ghelardini 2006). During lytic development, Mu gene expression is catalyzed by the host RNAP (Toussaint and Lecocq 1974) and occurs in three phases: early, middle, and late (Marrs and Howe 1990; Stoddard and Howe 1990). The middle promoter P_m_ contains a −10 hexamer, but lacks a −35 hexamer. Only 1 or 2 bases match the −35 consensus TTGACA even when allowing a broad range of 17 ± 4 bp spacing between the −10 and −35 hexamers. As P_m_ also lacks an extended −10 sequence (TGn just before the −10 hexamer) (Keilty and Rosenberg 1987; Barne et al. 1997), transcription initiation at P_m_ requires an activator, the early gene product Mor (Mathee and Howe 1990, 1993; Stoddard and Howe 1990). Both mutational analysis and DNase I footprinting showed that a Mor dimer binds to a dyad-symmetry element located upstream and overlapping the −35 region in P_m_ (Artsimovitch and Howe 1996; Artsimovitch et al. 1996). Addition of RNAP to a mixture of Mor and P_m_ DNA resulted in a typical RNAP DNase I footprint extending downstream from Mor, as well as a short upstream footprint caused by binding of the *α*CTD (Artsimovitch et al. 1996). In in vitro transcription assays, the *C*-terminal domains of both the *α* and *σ* subunits were required for efficient activation of P_m_ by Mor, leading to the model shown in Figure1 for the Mor-RNAP-P_m_ ternary complex (Artsimovitch et al. 1996).

Mu late transcription must be activated by the Mu C protein (Margolin et al. 1989; Stoddard and Howe 1989; Marrs and Howe 1990), which is closely related to Mor (Mathee and Howe 1990). The C protein binds to a dyad-symmetry element in the four late promoters (P_lys_, P_I_, P_P_, and P_mom_) that is related to the dyad-symmetry element in P_m_ (Chiang and Howe 1993; Artsimovitch and Howe 1996). Despite the relatedness of these proteins and promoters, their activities were highly specific. Mor did not activate the late promoters and conversely C did not activate P_m_ (Stoddard and Howe 1989; Marrs and Howe 1990; Mathee and Howe 1990). The P_mom_ promoter region is unusual; it contains two divergent promoters, *mom*P1 and *mom*P2 (Balke et al. 1992). In the absence of C, RNAP bound preferentially to *mom*P2 (Sun and Hattman 1998). In the presence of C, RNAP binding shifted from *mom*P2 to *mom*P1, increasing *mom* gene transcription from *mom*P1 and reducing transcription from *mom*P2 (Balke et al. 1992). Thus, one part of the activation mechanism by C at P_mom_ is repositioning of RNAP from *mom*P2 to *mom*P1 (Balke et al. 1992).

In this report, we present experiments which demonstrate that RNAP can interact with P_m_ in the absence of Mor, producing not a clear footprint, but instead two hypersensitive bands. Focusing on the band at promoter position −51, we show that this hypersensitive band occurs irrespective of the promoter length, incubation temperature, and open complex formation. It requires the complete RNAP including its *α*CTD; deletion of the *α*CTD prevents −51 hypersensitivity, and provision of only the *α*CTD or intact *α* also prevents hypersensitivity, indicating a role for other RNAP subunits in its origin. Strikingly, the intensity of the −51 hypersensitivity does not correlate with promoter activity. In an “order of addition” experiment, we show that preincubation of Mor with P_m_ DNA, followed by addition of RNAP, results in the most rapid open complex formation, whereas preincubation of RNAP with P_m_ DNA actually slows the formation of open complexes, arguing against the possibility that Mor might reposition a prebound RNAP, as C does at P_mom_. Therefore even though P_m_ and P_mom_ are quite similar, as are Mor and C, the mechanisms of transcriptional activation they mediate are quite distinct.

## Methods

### Chemicals, enzymes, and media

Standard chemicals for working with DNA and proteins were usually obtained from Sigma Chemical Co. (St. Louis, MO) and BioRad (Hercules, CA), respectively. Some buffer and media components were obtained from Fisher (Fair Lawn, NJ) or JT Baker (Phillipsburg, NJ). Sources for specific chemicals can be found in previous publications, including those by Artsimovitch and Howe (1996), Kahmeyer-Gabbe and Howe (1996), Kumaraswami et al. (2004), and Jiang and Howe (2008). Seakem ME and NuSieve GTG-agarose were from FMC Bioproducts (Philadelphia, PA); KMnO_4_ was from Aldrich Chemical Company (Milwaukee, WI); the Ni-NTA column was from Qiagen (Stanford, CA); and the Superdex 75 pg was from Amersham Biosciences (Piscataway, NJ). Isopropyl-*β*-D-thiogalactopyranoside (IPTG) and *ortho*-nitrophenyl-*β*-galact-opyranoside (ONPG) were from US Biological (Swampscott, MA) and American Biorganics, Inc. (Sanborn, NJ), respectively. Radiolabeled [γ-^32^P] ATP (3000 Ci/mmol) was from Perkin Elmer Life Science (Shelton, CT), and dNTPs were from Promega (Madison, WI). The DNase I (type II from bovine pancreas) was purchased from Sigma Chemical Co. Enzymes *Eco*RI and *Bam*HI were from New England Biolabs (Ipswich, MA); T4 polynucleotide kinase was from Promega. The *Taq* DNA polymerase, T4 DNA ligase and shrimp alkaline phosphatase were from Roche (Applied Science, Indianapolis, IN).

Minimal medium with casamino acids (M9CA; Kahmeyer-Gabbe and Howe 1996) was used in Mor overexpression and *β*-galactosidase assays. Standard LB (Miller 1972) was used for overexpression of the His-tagged *α* subunit and the His-tagged *α*-CTD. Modified LB (Howe 1973) containing only half as much NaCl was used for other cell growth purposes. Chloramphenicol (Cm) at 25 *μ*g/mL and ampicillin (Ap) at 40 *μ*g/mL were added to media when necessary for plasmid maintenance.

### Bacterial strains

The host strain background for most plasmid constructions and in vivo assays was *Escherichia coli* K-12 strain MH13312 (*mcrA Δpro-lac thi gyrA-96 endA-1 hsdR-17 relA-1 supE-44 recA* / F' *pro*^+^ *lacI*^*Q1*^ *ΔlacZY*), a derivative of JM109 carrying an F' plasmid deleted for both *lacZ* and *lacY* and expressing higher than normal levels of Lac repressor (Artsimovitch and Howe 1996). Strains MH13335 and MH13337 are derivatives of MH13312 containing only pKM78 or both pKM78 and pIA14, respectively. Strains MH15001, MH15751, and MH15754 are derivatives of MH13312 containing pKM78 and pMM1, pYM113, or pYM114, respectively. Strain MH12112 *F*^*+*^ *ΔaraD*-*leu*::Mu *c*ts61 *zai*-*737*::Tn10 Δ*lac* Tet^R^/pMK100) was used as a source of plasmid pMK100 (Kahmeyer-Gabbe and Howe 1996). Strain MH10713, a derivative of strain BL21 (*E.coli* B *ompT* r_b_^−^ m_b_^−^ λDE3 pLysS) was freshly transformed with plasmid pKM90 and used for Mor protein overexpression (Artsimovitch and Howe 1996).

### Plasmids and plasmid construction

Plasmids used for in vivo promoter activity assays were multistep derivatives of the promoter cloning vector pRS415 (Simons et al. 1987). Plasmid pRS415 contains a pBR322 replicon, an *Eco*RI-*Sma*I-*Bam*HI polylinker upstream of a promoter-less *lac* operon and five tandem repeats of the *rrnB* transcription terminator upstream of the polylinker to prevent expression of *lacZ* by read-through of plasmid transcripts. The *lacY* gene was deleted to generate pLC1 (Chiang and Howe 1993) and a 20-bp *Hin*dIII linker was cloned just upstream of the polylinker in pLC1 to generate pIA12 (Artsimovitch and Howe 1996). Different lengths of P_m_ promoter DNA were amplified by PCR using oligonucleotide primers containing *Eco*RI (upstream primer) and *Bam*HI (downstream primer) sites and cloned into *Eco*RI-*Bam*HI-digested pIA12 to generate the following P_m_-*lacZ* fusion plasmids: pYM114 contains P_m_ sequences from −98 to +46, pMM1 has P_m_ −98 to +10, pYM113 has P_m_ −62 to +46. Plasmid pIA14 has P_m_ −62 to +10 cloned into pIA12 (Artsimovitch and Howe 1996), and plasmid pMK100 contains P_m_ sequences from −198 to +146 cloned into pLC1 (Kahmeyer-Gabbe and Howe 1996). The sequences of the above promoter fragments were confirmed by dideoxy-sequence analysis (Sanger et al. 1977) using primers IRI21 and/or IRI22 which are homologous to vector sequences flanking the polylinker (Artsimovitch and Howe 1996).

Plasmid pKM78 (Mathee and Howe 1990) contains a P_*lacUV5*_-*mor* operon fusion and *lacI*^*q*^ gene cloned into a plasmid containing a P15A replicon and encoding chloramphenicol resistance. Cells containing pKM78 were induced with 2 mmol/L IPTG to provide roughly physiological levels of Mor protein for in vivo P_m_-*lacZ* promoter activity assays. Plasmid pKM90 contains the *mor* gene under T7 promoter control and located between the *Nde*I and *Bam*HI sites of pT7-7, which has a ColE1 replicon and confers Ap resistance (Mathee and Howe 1993); it was used for overexpression of Mor for purification.

### *β*-galactosidase assays

The *β*-galactosidase assays for P_m_ activity in the presence and absence of Mor were performed as described by Miller (1972) with minor modifications (Chiang and Howe 1993). Enzyme activities were calculated according to Miller (1972) and normalized to the activity of a control culture containing wild-type P_m_ (−98 to +46) that was assayed in the same experiment, setting the wild-type activity to 1000 units. The activities presented were derived by averaging the results obtained in at least three independent assays.

### Proteins

Mor overexpression and purification was performed as described by Artsimovitch and Howe (1996) except that ammonium sulfate was used at a concentration of 20% instead of 23%. Purified RNAP was a generous gift from Ding J. Jin. Purified His-*α*CTD and thrombin-cleaved untagged *α*CTD were thoughtfully provided by Muthiah Kumaraswami. Purified His-*α* protein was gratefully received from Ji Ma. Uncleaved His-*α*CTD contained 21 extra amino acids at its N-terminus; thrombin-cleaved *α*CTD has only four extra N-terminal amino acids. The reconstituted wild-type and mutant (Δ*α*CTD) RNA polymerases were graciously provided by Wilma Ross and Richard Gourse.

### DNase I footprinting

The top strand primer was 5′ end-labeled by treatment with T4 polynucleotide kinase and [γ-^32^P] ATP (3000 Ci/mmol); the enzyme was heat-inactivated, and the mixture was added directly to a PCR reaction containing an unlabeled opposing primer and plasmid DNA template containing wild-type or mutant P_m_. Varying amounts of purified Mor and/or RNAP were incubated with 0.42 nmol/L probe in DNase I footprinting binding buffer (25 mmol/L Tris-HCl [pH 7.5], 50 mmol/L NaCl, 0.5 mmol/L MgCl_2_, 2 mmol/L CaCl_2_, 0.5 mmol/L EDTA, 1 mmol/L DTT, 7% glycerol, 1 ng calf thymus DNA/*μ*L) at 30**°**C for 20 min in a 40-*μ*L reaction volume. Next DNase I (4.5 ng) was added to the binding reactions which were then incubated for 1 min at room temperature and stopped by addition of 50 *μ*L of stop solution (200 mmol/L NaCl, 250 *μ*g tRNA/mL, 10 mmol/L EDTA, 1% SDS). The DNase I treated samples were subjected to phenol:chloroform extraction (Artsimovitch et al. 1996) and ethanol precipitation (Artsimovitch et al. 1996), dried, resuspended in standard loading buffer (Sambrook et al. 1989) and subjected to electrophoresis in a 6% sequencing gel (Artsimovitch et al. 1996). Markers used in several figures were generated by performing a G-only Maxam–Gilbert sequencing reaction (Maxam and Gilbert 1980) with the same end-labeled probe. The DNA fragments were visualized by autoradiography on Kodak BioMS film (Kodak Corp., Rochester, NY) with an intensifying screen.

### KMnO_4_ footprinting

Bottom strand-labeled probe was made as described above for DNase I footprinting except that the 5′ end of the bottom strand primer was labeled. The probe was incubated in DNase I binding buffer lacking CaCl_2_, with different amounts of purified Mor, RNAP, or both in a 40-*μ*L volume. Mor was added 5 min before RNAP unless stated otherwise. After 15 min of incubation at the desired temperature, each reaction mixture received 3 *μ*L of freshly prepared 37.5 mmol/L KMnO_4_ and was incubated for 1 min, then each reaction was stopped by addition of 5 *μ*L *β*-mercaptoethanol and 150 *μ*L 30 mmol/L EDTA. After extraction with 200 *μ*L phenol:chloroform (25:24) to remove the proteins, 60 *μ*L of Quench A solution (3 mol/L NH_4_CH_3_COOH, 1 mol/L *β*-mercaptoethanol, 250 *μ*g tRNA/mL, 20 mmol/L EDTA) and 650 *μ*L of cold absolute ethanol were added to each tube and the mixtures were held on dry ice for 15 min for DNA precipitation. Following centrifugation, each DNA pellet was washed once with cold 70% ethanol and dried in a SpeedVac rotary evaporator. The modified DNAs were cleaved by treatment with 70 *μ*L of a freshly made 10% piperidine solution at 90°C for 30 min. The DNA samples were dried, and the remaining piperidine was removed by SpeedVac drying following sequential resuspension in 30 and 20 *μ*L water. The samples were subjected to electrophoresis on a 6% sequencing gel (Sambrook et al. 1989) and visualized by autoradiography on Kodak BioMS film with a Kodak BioMaxMS intensifying screen.

## Results and Discussion

In previous DNase I footprinting experiments with P_m_, when RNAP was present and Mor absent, hypersensitive sites (HS) were noted, suggesting that RNAP potentially interacted with P_m_ in the absence of Mor (Artsimovitch et al. 1996). Such interaction was observed previously at P_mom_, where one role of C was to reposition prebound RNAP from *mom*P2 to *mom*P1, thereby increasing transcription of *mom*. Experiments described in this report were designed to test (1) whether RNAP interaction with P_m_ in the absence of Mor plays a role in activation of P_m_ in the presence of Mor_._ (2) whether or not Mor repositions a prebound RNAP at P_m_, and (3) whether evidence for a role of Mor in recruitment of RNAP could be detected by a time difference for open complex formation when two of the three interacting components—RNAP, Mor and P_m_—were preincubated together and then the third added last.

**Figure 2:**
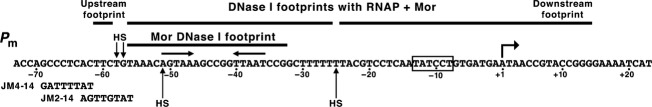
The P_m_ sequence with DNase I footprints. The sequence of P_m_ from −73 to +23 is shown with dots indicating 10-base intervals that are assigned “−” numbers upstream and “+” numbers downstream of +1, the initiation site. The bars indicate the bases protected from DNase I digestion by the proteins shown. Inverted arrows correspond to the position of the dyad-symmetric Mor binding site; vertical arrows indicate the locations of hypersensitive sites (HS) cleaved by DNase I; the −10 hexamer is in a box, and the bent arrow designates the start of the RNA transcript at +1. The altered sequences present in two mutants, JM2-14 and JM4-14, are aligned directly below the corresponding positions in P_m_.

To assist the reader in following and understanding the results of the footprinting experiments to be presented, we have included Figure2 which contains the P_m_ DNA sequence annotated with the positions of the dyad-symmetry element for Mor binding (−51 to −36). Figure2 also shows the locations of DNase I footprints produced by Mor alone (−56 to −36) and by Mor and RNAP together (−56 to +14 and −61 to −59), as well as the locations of multiple hypersensitive sites. The position of the −10 hexamer is also indicated, but no −35 sequence is marked. In contrast to many activator-dependent promoters (Salgado et al. 2013), there are simply too few base matches (only 1 or 2) in the −35 region of P_m_ with the −35 consensus sequence (TTGACA), even when tested with spacings of 17 ± 4 between the −10 and possible −35 sequences, to identify a candidate −35. We note that P_m_ also lacks the extended −10 sequence, TGn, located immediately upstream of −10 that allows transcription in the absence of a −35 element (Keilty and Rosenberg 1987; Barne et al. 1997).

### Hypersensitive bands produced by RNAP in the absence of Mor indicate an interaction between RNAP and P_m_

**Figure 3:**
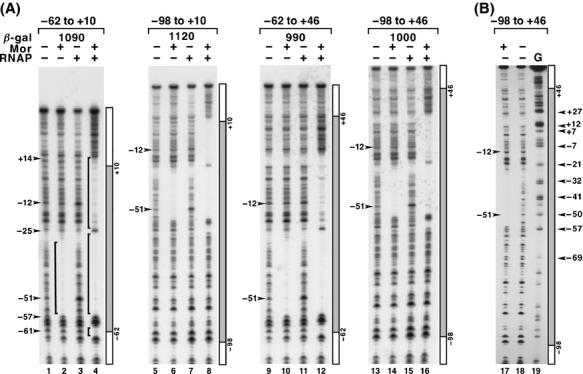
DNase I footprinting of probes containing different lengths of wild-type P_m_ DNA. Panel (A) contains linear, top strand, 5' end-labeled DNA fragments (0.42 nmol/L) containing different lengths of P_m_ DNA (shaded bar) and flanking plasmid vector DNA (open bar) were incubated with Mor (800 nmol/L) and/or RNAP (56 nmol/L) at 30°C for 20 min, then treated with DNase I as described in Methods. When both Mor and RNAP were used, Mor was added to DNA first and the reaction was incubated for 5 min prior to addition of RNAP. The length of P_m_ DNA in the probe is given above the bracket for each panel. The relative *β*-galactosidase (*β*-gal) activity produced in vivo by a P_m_-*lacZ* fusion plasmid carrying those sequences is given just below the bracket and is the average derived from three independent assays. The presence or absence of each protein is indicated by a “+” or “−”, respectively, above each lane. The positions of relevant bands are indicated by arrowheads to the left of lane 1. The bar to the left of lane 2 identifies the Mor footprint, whereas those to the left of lane 4 indicate footprints arising in the presence of both Mor and RNAP. Panel (B) contains DNA treated as in panel (A) with an additional lane containing a G-ladder and was derived from a different experiment. Lane 19 contains a G-ladder with arrowheads marking the promoter positions of specific G-ladder bands. These bands migrate 1.5 nucleotides faster than corresponding DNase I fragments (Sollner-Webb and Reeder 1979).

In previous DNase I footprinting experiments using P_m_ sequences −62 to +10, we noted the absence of a footprint, but the presence of several hypersensitive bands when RNAP and P_m_ DNA were mixed in the absence of Mor (Artsimovitch et al. 1996). As prebound RNAP might influence the mechanism by which Mor activates P_m,_ as it does for C at P_mom_ (Balke et al. 1992), we decided to examine RNAP binding to P_m_ and the origin and role of −51 hypersensitivity in more detail. First, we compared the band patterns generated by addition of Mor alone, RNAP alone, and both proteins together with those produced in a “no protein” control. These binding reactions were performed at 30°C to allow comparison of the footprint patterns with those of open complexes produced by Mor and RNAP together. The band patterns showed that Mor alone protected a region from −56 to −33 (Fig.3, lane 2); whereas Mor and RNAP together protected a region from −61 to +14 except for positions −25, −57, and −58, which remained accessible and somewhat hypersensitive to DNase I cleavage (Fig.3, lane 4). RNAP alone gave little protection but caused bands at positions −12 and −51 to become hypersensitive, with that at −51 exhibiting the greater effect (Fig.3, lane 3). These results indicated that RNAP could interact with P_m_ DNA in the absence of Mor.

### Hypersensitivity at −51 is not influenced by vector sequence

To test the effect of flanking vector sequence on RNAP binding in the absence of Mor, we carried out DNase I footprinting reactions with probes containing P_m_ sequences −98 to +10, −62 to +46, and −98 to +46 (Fig.3). The Mor footprints and open complex footprints were the same as those observed with probe −62 to +10 regardless of the promoter length (Fig.3, even-numbered lanes). For reactions with RNAP but without Mor, there was little if any reduction in hypersensitivity at position −51 in the probes extended to −98 relative to those ending at −62 and those extended to +46 relative to +10 (Fig.3). As substantial hypersensitivity remained at position −51 regardless of promoter length, we chose to use it as the most sensitive indicator of RNAP association with P_m_ in the absence of Mor. When the above different length promoters were cloned into the P_m_-*lacZ* fusion vector and assayed for Mor-dependent promoter activity, they gave a range of *β*-galactosidase activities (an average from three assays) between 990 and 1120 units relative to promoter −98 to +46 which was arbitrarily set to 1000 units (Fig.3). These differences are within the range of *β*-galactosidase values obtained for the same promoter assayed independently multiple times (Chiang and Howe 1993) and therefore are unlikely to be significant. We concluded from these results that this interaction was a natural feature of P_m_ and, therefore, might play a mechanistic role in P_m_ activation.

### Hypersensitivity at −51 is observed at 5°C, 15°C, and 30°C

**Figure 4:**
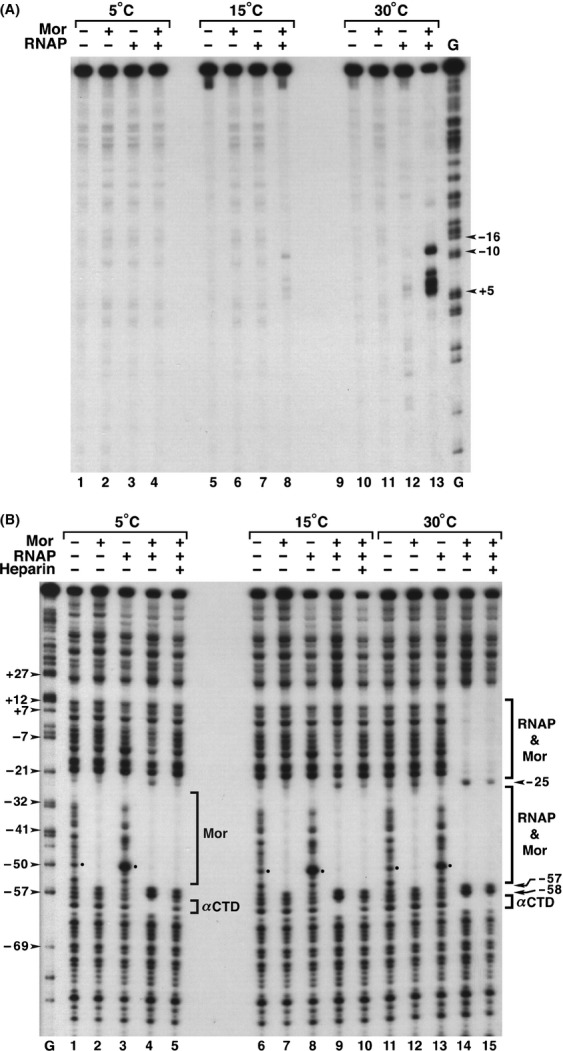
DNase I and KMnO_4_ footprinting at different temperatures. Binding reactions were generated with probe containing P_m_ sequence −98 to +46 and flanking vector DNA, as described for Figure3 except that samples were incubated at 5°C, 15°C, and 30°C. For KMnO_4_ footprinting in panel (A) the probe was labeled at the 5′ end of the bottom strand. After 5 min incubation with Mor and 6 min incubation with RNAP, the samples were subjected to KMnO_4_ modification and cleavage. Arrowheads mark promoter positions of specific G-ladder bands. For DNase I footprinting in panel (B) lanes 5, 10, and 15 also received heparin to 100 ng*/μ*L, and the mixture was incubated for 1 min prior to DNase I digestion. Bars indicate the extent of the footprints generated by Mor alone, or by Mor and RNAP together. Arrowheads indicate the promoter positions of G-ladder bands. Dots mark the bands at position −51. Arrows and arrowheads marked HS identify positions −25, −57, −58 that are hypersensitive to DNase I digestion.

Interactions of RNA polymerase with promoters can vary with temperature (Cowing et al. 1989; Schickor et al. 1990; Mecsas et al. 1991), for example, the transition from closed to open complexes requires temperatures above 20°C. Therefore, we investigated the interactions of RNAP with P_m_ in the absence of Mor over a range of temperatures. Figure4A shows that P_m_ also follows this general rule. In the presence of both Mor and RNAP open complex formation, assayed by permanganate footprinting, occurred well at 30°C (Fig.4A, lane 13), not at all at 5°C (Fig.4A, lane 4), and extremely poorly, if at all, at 15°C (Fig.4A, lane 8). To examine the temperature dependence of the −51 hypersensitivity, we carried out DNase I footprinting at 5°C, 15°C, and 30°C so we could compare the band patterns at low temperature to those of open complexes produced at 30°C. Binding of Mor alone, as assayed by Mor footprint formation, occurred equally well at all three temperatures (Fig.4B, lanes 2, 7, and 12). Binding of RNAP in the absence of Mor, as assayed by position −51 hypersensitivity, also occurred well at all three temperatures (Fig.4B, dots identify position −51 in lanes 3, 8, and 13). As expected, incubation of Mor and RNAP with P_m_ DNA at 5°C and 15°C did not produce the RNAP-dependent downstream footprints from −34 to +14 characteristic of open complexes; whereas heparin-resistant open complexes were formed in reactions incubated at 30°C (Fig.4B, lanes 14 and 15). Note that the RNAP *α*CTD footprints from −59 to −61 upstream of bound Mor were generated equally well at all three temperatures (Fig.4B, lanes 4, 9, and 14), indicating that they arose independently of open complex formation, and most likely reflected the presence of closed complexes formed at 5°C and 15°C. The upstream footprints observed at 5°C and 15°C were abolished by addition of heparin (Fig.4B, lanes 5 and 10), supporting the hypothesis that they reflected the presence of closed complexes. In contrast, the Mor footprint, and therefore Mor binding, was unaffected by the addition of heparin (Fig.4B, lanes 5 and 10). The presence of the upstream footprint in the absence of heparin at 5°C and 15°C demonstrated that RNAP could bind to P_m_ in the presence of Mor, even in the absence of open complex formation (Fig.4B, lanes 4 and 9). With RNAP alone, the −51 hypersensitivity occurred at all three temperatures, and thus, is Mor-independent and does not require open complex formation. We propose that it arises by a transient interaction of RNAP with P_m_. In contrast, when both RNAP and Mor were present, the −51 hypersensitivity was prevented by Mor binding; instead a footprint just upstream of Mor arose at all three temperatures, which we conclude is due to the presence of heparin-sensitive closed complexes at 5°C and 15°C and heparin-resistant open complexes at 30°C. The clarity of the upstream footprint, presumably caused by *α*CTD binding, demonstrated that RNAP binding in the presence of Mor was quite strong, in essence we propose, using Mor-RNAP interactions to tether RNAP to the promoter in the absence of stable RNAP −10 interaction.

### Deletion of the *α*CTD from RNAP prevents −51 hypersensitivity but other RNAP subunits are also required

**Figure 5:**
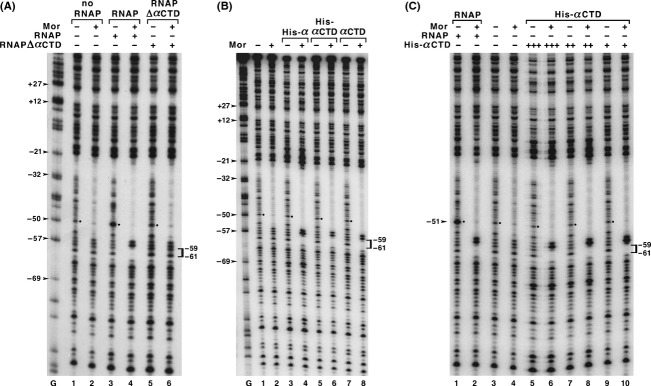
DNase I footprinting with RNA polymerases and different forms of the *α*CTD at 5°C. The DNA probe was linear, top strand and 5′ end-labeled with P_m_ sequences from −98 to +46 and flanking plasmid vector DNA. (A) The probe was preincubated with or without Mor (800 nmol/L) for 5 min, then RNAP or RNAP-Δ*α*CTD was added and the reactions incubated for 20 min prior to DNase I digestion. The presence and absence of the proteins are designated by “+” and “−” signs above each lane. The upstream footprint is identified with a bracket, and dots mark the bands for position −51. Arrowheads mark the positions of G ladder bands, which migrate 1.5 nucleotides faster than bands generated by DNase I cleavage (Sollner-Webb and Reeder 1979; Artsimovitch et al. 1996). (B) The same DNA probe was incubated with His-*α* (9 *μ*mol/L), His-*α*CTD (35 *μ*mol/L), and *α*CTD (35 *μ*mol/L) at 5°C for 20 min prior to DNase I digestion. Labeling follows that in panel (A). (C) The same DNA probe was incubated with (+) and without (−) Mor (800 nmol/L) and/or His-*α*CTD at 35 *μ*mol/L (+), 175 *μ*mol/L (++), and 350 *μ*mol/L (+++) or RNAP (56 nmol/L) prior to treatment with DNase I. The positions of band −51 are identified on the left, and the positions of the −59 to −61 upstream footprint are identified on the right.

We knew from previous experiments that (1) binding of the *α*CTD to P_m_ resulted in a small footprint upstream of Mor and (2) Mor and *α*CTD bound synergistically to P_m_ without *α*NTD or any other subunit of RNAP. To ask whether the −51 hypersensitivity would display the same properties, we carried out DNase I footprinting at 5°C with reconstituted RNA polymerases containing and lacking *α*CTD (Fig.5A). Complete RNAP alone yielded −51 hypersensitivity (Fig.5A, lane 3); whereas, in the presence of Mor, RNAP addition generated the usual upstream footprint but no −51 hypersensitivity (Fig.5A, lanes 3 and 4). In contrast, when RNAP lacking *α*CTD (RNAP Δ*α*CTD) was used, there was neither an upstream footprint nor −51 hypersensitivity (Fig.5A, lanes 5 and 6), showing that the *α*CTD plays an important role in the association of RNAP with P_m_ irrespective of the presence or absence of Mor. When we carried out DNase I footprinting with purified His-*α*, His-*α*CTD and *α*CTD in the absence of Mor, neither an upstream footprint nor −51 hypersensitivity was observed (Fig.5B, lanes 3, 5, and 7). In the presence of Mor, all three proteins (His-*α*, His-*α*CTD, and *α*CTD) bound and produced upstream footprints, but no −51 hypersensitivity (Fig.5B, lanes 4, 6, and 8). To determine whether the His-*α*CTD could bind to the upstream region without Mor, we performed DNase I footprinting with increasing concentrations of the His-*α*CTD, up to 10 times the normal amount (Fig.5C). In the absence of Mor, there was no upstream footprint or −51 hypersensitivity, even at concentrations of His-*α*CTD so high that binding to other AT-rich regions was detected (Fig.5C, lane 5) In the presence of Mor, there was complete protection at −61 to −59 even at low His-*α*CTD concentrations (Fig.5C, lane 10), and the upstream footprint observed was the same as that caused by RNAP (Fig.5C, lane 2). These results demonstrated that additional subunits of RNAP were required to form the −51 hypersensitivity, a result just the opposite of that for the upstream footprint. These results also indicated that Mor-*α*CTD interactions provided the driving force for stable *α*CTD binding to the upstream UP-like element.

### The intensity of −51 hypersensitivity does not correlate with promoter activity

**Figure 6:**
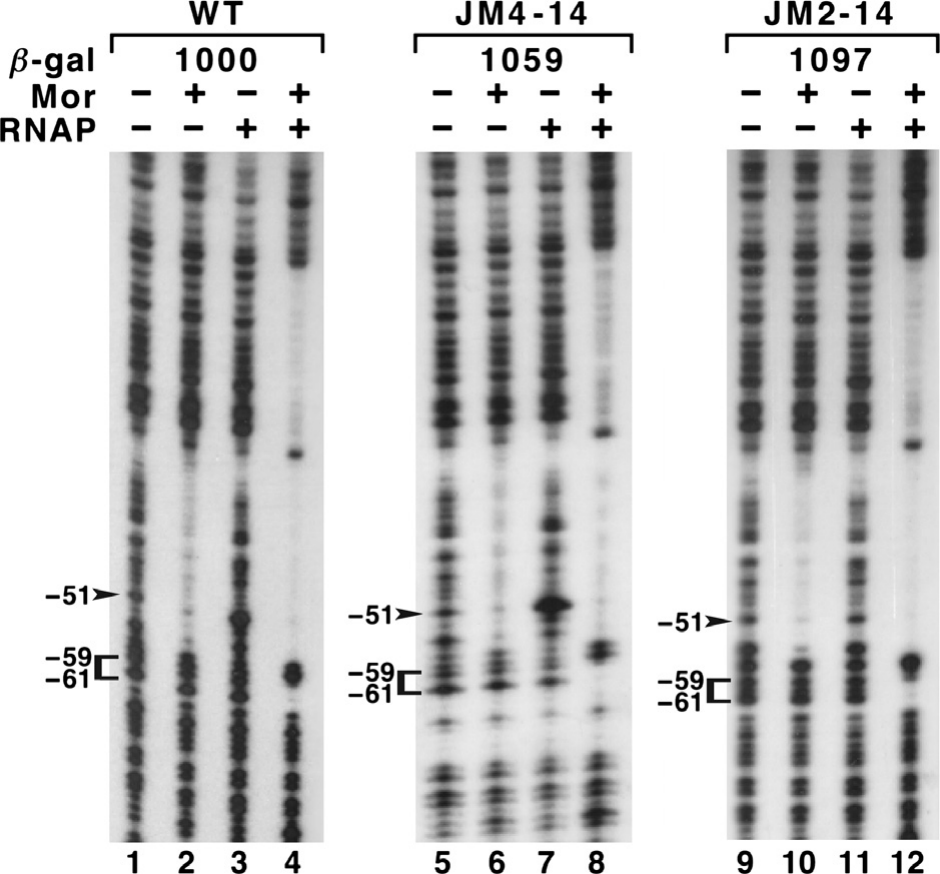
DNase I footprinting with wild-type and mutant P_m_ probes. All probe DNAs were 5′-end labeled and contained P_m_ sequences from −98 to +10 and flanking plasmid vector DNA. Probes JM4-14 and JM2-14 contain the mutations shown in Figure2. Binding reactions were performed and the figure labeled as described for Figure3.

The above experiments demonstrated that both the *α*CTD and part or all of the remainder of RNAP were required to generate the −51 hypersensitivity, but they did not address the role of the −51 hypersensitivity in P_m_ activation. If activation of P_m_ were caused by a Mor-dependent repositioning of a prebound RNAP, one would expect to see a correlation between promoter activity and the degree of hypersensitivity at position −51. We tested this hypothesis by assaying for position −51 hypersensitivity in a pre-existing collection of mutants with base changes upstream of −57. These mutants had essentially wild-type *β*-galactosidase activities, but a wide range of −51 hypersensitivities. DNase I footprinting was carried out for two mutants with three (JM2-14) or five (JM4-14) base changes in the region from −68 to −57 (Fig.2) in the context of a P_m_ clone with P_m_ sequences from −98 to +10. The DNase I footprint patterns for these mutants and the −98 to +10 wild-type probe illustrate the range of hypersensitivities observed (Fig.6) The Mor footprints and open complex footprints were essentially identical for the wild-type and mutant DNAs. In contrast, in lanes with probe and RNAP alone, the position −51 hypersensitivity was dramatically increased for one mutant (JM4-14; lane 7; *β*-galactosidase value: 1059 units) and greatly decreased for the other (JM2-14; lane 1; *β*-galactosidase value: 1097 units). These results demonstrated that there was no correlation between the levels of position −51 hypersensitivity in the absence of Mor and promoter activity in the presence of Mor. This argued against Mor repositioning prebound RNAP.

### Order of addition experiments showed the fastest footprints with Mor prebound to P_m_

**Figure 7:**
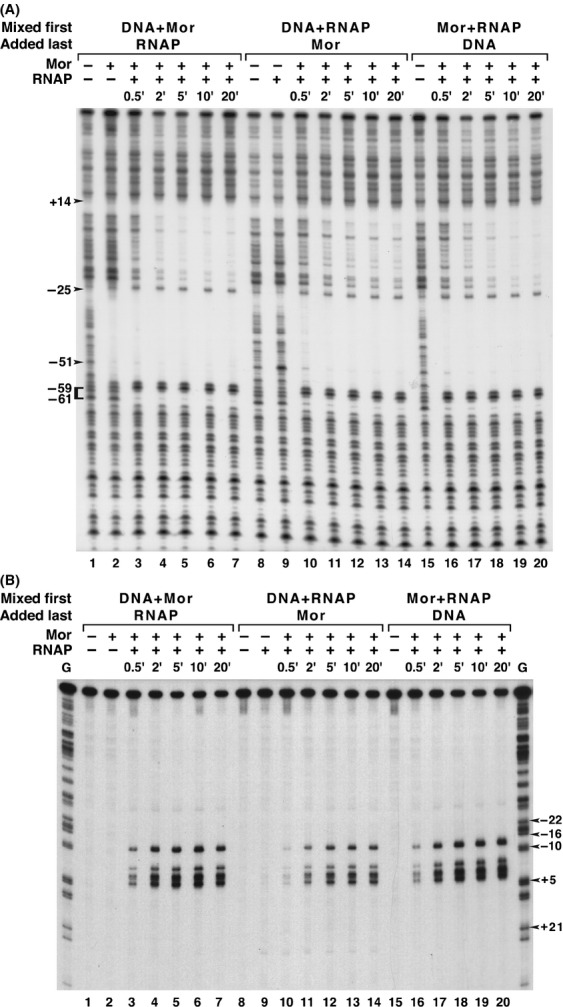
Order of addition DNase I and KMnO_4_ footprinting. The probe contained wild-type P_m_ sequence from −98 to +46 (and flanking plasmid vector DNA). For DNase I digestions, the DNA probe was labeled at the 5′ end of the top strand. For KMnO_4_ footprinting, the DNA probe was labeled at the 5′ end of the bottom strand. The three sets of reactions differed in which two components were incubated together (Mixed first) prior to addition of the third component (Added last). The amounts of each protein and probe used were the same as for Figure3. After addition of the third component, samples were subjected to DNase I digestion (A) or KMnO_4_ treatment and cleavage (B) at the times indicated above each lane. In the left set of reactions (lanes 1–7), Mor was preincubated with the P_m_ probe for 5 min before RNAP addition; in the middle set (lanes 8–14), RNAP was preincubated with the probe for 6 min before Mor addition; in the right set (lanes 15–20), Mor and RNAP were preincubated together for 6 min before addition of P_m_ probe. The positions of the hypersensitive band at −51 and the upstream footprint are indicated. Arrowheads mark the promoter positions of specific G-ladder bands.

Theoretically, there are multiple possible sequential interactions that could lead to Mor-dependent P_m_ activation: (1) Mor might recognize and bind to P_m_, then recruit RNAP to bind and initiate transcription; (2) RNAP might be continually bound to P_m_ with Mor functioning at one or more “post-recruitment” steps, such as isomerization or promoter clearance; or (3) RNAP and Mor might form a complex in solution before binding to P_m_ DNA. To determine which of these possibilities was most likely, we conducted an “order of addition” experiment, asking whether preincubation of any two of the three components (Mor, RNAP, and P_m_ DNA) would lead to faster open complex formation following addition of the third component. All three possible orders of addition were tested. In the first experiment, open complexes were assayed by the generation of an RNAP-dependent DNase I footprint in the region from −23 to +14 (Fig.7A). When Mor was bound to P_m_ DNA first, addition of RNAP resulted in the fastest footprint generation, with maximal protection achieved within 5 min (Fig.7A, lanes 1–7). When RNAP was preincubated with Mor followed by addition of P_m_ DNA last, the 5 min footprint was less clear and maximal protection was first observed in the 10 min sample (Fig.7A, lanes 15–20). When RNAP was preincubated with P_m_ DNA and Mor added last, it took even longer for a footprint to form and complete protection was never achieved, even after 20 min of incubation following Mor addition (Fig.7A, lanes 8–14). Thus, it appeared that prebinding of RNAP delayed rather than assisted open complex formation.

As monitoring the loss of signal, as in a DNase I footprint, is inherently less sensitive than the positive generation of a signal over a clear background, we repeated the experiment using KMnO_4_ footprinting to monitor the generation of permanganate-sensitive, single-stranded DNA. As expected, T residues on the bottom strand at positions −12, −4, −1, +1, +3, and +4 were permanganate sensitive (Fig.7B, lanes 16–20). Again, prebinding of Mor to P_m_ DNA gave the most rapid open complex formation (Fig.7B, lane 3); whereas prebinding of RNAP to P_m_ gave the slowest; it took 2 min to reach the same signal intensity that was observed in the 0.5 min sample with Mor prebound to P_m_ DNA (Fig.7B, lane 11). Although the time dependence of footprint formation was not dramatically different, it was reproducible and observed with both DNase I and KMnO_4_ footprinting assays. These results supported the contention that Mor binds first and recruits RNAP to the promoter and argue against the possibility that Mor repositions a bound RNAP. Based on the similarity between Mor and C, one might have a priori expected Mor and C to have similar activation mechanisms, but just as the differences between CAP protein function at different promoters, they did not.

## Summary

These results answer three important questions: (1) They demonstrated that −51 hypersensitivity arose from interaction of RNAP and P_m_ in the absence of Mor. This interaction was not altered by promoter length, vector sequences or incubation temperature, indicating that it is a natural feature of this promoter. It required the complete RNAP and did not occur with an RNAP lacking its *α*CTDs. It was not produced by *α* or the *α*CTD alone, indicating that one or more of the other subunits of RNAP were also involved. These are exactly opposite the requirements for the upstream footprint produced in the presence of Mor, in which Mor and the *α*CTD were the only proteins needed to produce it. These results demonstrated that the *α*CTD plays an important role in association of RNAP with P_m_ in both the presence and absence of Mor. Furthermore, they also indicated that Mor-*α*CTD interactions provided the driving force for stable *α*CTD binding to the upstream UP-like element, in essence tethering the RNAP to P_m_ in closed complexes. As there was no correlation between the intensity of the −51 hypersensitivity and promoter activity, we were unable to demonstrate that it plays a role in the activation of transcription in the presence of Mor. (2) Our data make it highly unlikely that Mor repositions a prebound RNAP. First, a higher degree of hypersensitivity, indicative of greater binding of RNAP in the absence of Mor, did not increase promoter activity in the presence of Mor, arguing against a role for Mor repositioning RNAP. Second, the observation in the “order of addition” experiment was that the presence of RNAP bound to P_m_ actually delayed open complex formation relative to the other orders. (3) In the “order of addition” experiment, the three possible orders showed significant differences in the timing of open complex formation. The order with Mor binding to P_m_ first produced open complexes the most quickly, supporting the hypothesis that Mor recruits RNAP to the promoter.

These results notably increase our knowledge regarding transcription activation by Mor at P_m_. In addition, they demonstrate the considerable strength of Mor interaction with the *α*CTD of RNA polymerase, enough to tether the RNAP to the promoter in the absence of open complex formation. Finally, they have demonstrated that an “order of addition” experiment can effectively detect differences in the timing of open complex formation when they are large enough to contribute to investigations of transcriptional activation mechanisms.
